# Cognitive and emotional alterations in *App* knock-in mouse models of Aβ amyloidosis

**DOI:** 10.1186/s12868-018-0446-8

**Published:** 2018-07-28

**Authors:** Yasufumi Sakakibara, Michiko Sekiya, Takashi Saito, Takaomi C. Saido, Koichi M. Iijima

**Affiliations:** 1 Department of Alzheimer’s Disease Research, Center for Development of Advanced Medicine for Dementia, National Center for Geriatrics and Gerontology, Obu, Aichi 474-8511 Japan; 2 Laboratory for Proteolytic Neuroscience, RIKEN Center for Brain Science, Wako, Saitama 351-0198 Japan; 3 Department of Experimental Gerontology, Graduate School of Pharmaceutical Sciences, Nagoya City University, Nagoya, 467-8603 Japan

**Keywords:** Alzheimer’s disease, Amyloid precursor protein, Knock-in mouse model, Emotional behavior, Spatial learning

## Abstract

**Background:**

Alzheimer’s disease (AD), the most common cause of dementia, is characterized by the progressive deposition of amyloid-β (Aβ) peptides and neurofibrillary tangles. Mouse models of Aβ amyloidosis generated by knock-in (KI) of a humanized Aβ sequence provide distinct advantages over traditional transgenic models that rely on overexpression of amyloid precursor protein (APP). In *App*-KI mice, three familial AD-associated mutations were introduced into the endogenous mouse *App* locus to recapitulate Aβ pathology observed in AD: the Swedish (NL) mutation, which elevates total Aβ production; the Beyreuther/Iberian (F) mutation, which increases the Aβ42/Aβ40 ratio; and the Arctic (G) mutation, which promotes Aβ aggregation. *App*^*NL*-*G*-*F*^ mice harbor all three mutations and develop progressive Aβ amyloidosis and neuroinflammatory response in broader brain areas, whereas *App*^*NL*^ mice carrying only the Swedish mutation exhibit no overt AD-related pathological changes. To identify behavioral alterations associated with Aβ pathology, we assessed emotional and cognitive domains of *App*^*NL*-*G*-*F*^ and *App*^*NL*^ mice at different time points, using the elevated plus maze, contextual fear conditioning, and Barnes maze tasks.

**Results:**

Assessments of emotional domains revealed that, in comparison with wild-type (WT) C57BL/6J mice, *App*^*NL*-*G*-*F/NL*-*G*-*F*^ mice exhibited anxiolytic-like behavior that was detectable from 6 months of age. By contrast, *App*^*NL/NL*^ mice exhibited anxiogenic-like behavior from 15 months of age. In the contextual fear conditioning task, both *App*^*NL/NL*^ and *App*^*NL*-*G*-*F/NL*-*G*-*F*^ mice exhibited intact learning and memory up to 15–18 months of age, whereas *App*^*NL*-*G*-*F/NL*-*G*-*F*^ mice exhibited hyper-reactivity to painful stimuli. In the Barnes maze task, *App*^*NL*-*G*-*F/NL*-*G*-*F*^ mice exhibited a subtle decline in spatial learning ability at 8 months of age, but retained normal memory functions.

**Conclusion:**

*App*^*NL/NL*^ and *App*^*NL*-*G*-*F/NL*-*G*-*F*^ mice exhibit behavioral changes associated with non-cognitive, emotional domains before the onset of definitive cognitive deficits. Our observations consistently indicate that *App*^*NL*-*G*-*F/NL*-*G*-*F*^ mice represent a model for preclinical AD. These mice are useful for the study of AD prevention rather than treatment after neurodegeneration.

**Electronic supplementary material:**

The online version of this article (10.1186/s12868-018-0446-8) contains supplementary material, which is available to authorized users.

## Background

Alzheimer’s disease (AD) is characterized by a progressive decline in cognitive functions, usually starting with memory complaints, and eventually leading to multiple cognitive, neuropsychological, and behavioral deficits [1, 2]. The neuropathology of AD begins before overt cognitive symptoms, including the accumulation of amyloid-β peptide (Aβ) as extracellular plaques, aggregation of hyperphosphorylated tau as intracellular neurofibrillary tangles (NFTs), and activation of multiple neuroinflammatory pathways [3–5]. These brain pathologies are thought to induce neuronal cell loss in the hippocampus and cerebral cortex [3–5]. However, the etiology of AD is still not fully clarified, and many fundamental questions remain unanswered.

Mouse models of AD pathology are critical research tools for testing potential therapeutic approaches to AD and investigating the molecular mechanisms underlying AD pathogenesis [6, 7]. Several transgenic mouse lines overexpressing amyloid precursor protein (APP) have been developed as experimental models for Aβ amyloidosis [8, 9]. However, non-physiological overexpression of APP results in overproduction of various APP fragments in addition to Aβ [8], making it technically difficult to distinguish the pathophysiological effects caused by Aβ from those caused by other APP fragments [8, 10, 11]. Moreover, overexpression of APP causes memory impairment without Aβ deposition in some *App* transgenic mice [8, 11], suggesting that the brains of these transgenic mouse models may have pathophysiological properties that are not relevant to AD pathogenesis.

To produce Aβ pathology without non-physiological overexpression of APP in the mouse brain, alternative mouse models have been generated utilizing an *App* knock-in (KI) strategy [12] in which the murine Aβ sequence was humanized by changing three amino acids that differ between the mouse and human proteins. In addition, three familial AD-associated mutations were introduced into the endogenous mouse *App* locus: the Swedish (NL) mutation, which elevates total Aβ production [13]; the Beyreuther/Iberian (F) mutation, which increases the Aβ42/Aβ40 ratio [14, 15]; and the Arctic (G) mutation, which promotes Aβ aggregation [16, 17]. In *App*^*NL*-*G*-*F*^ mice, which harbor all three mutations within the Aβ sequence, Aβ amyloidosis is aggressively accelerated and neuroinflammation is observed in subcortical structures and cortical regions [12, 18]. By contrast, *App*^*NL*^ mice that carry only the Swedish mutation produce significantly higher levels of Aβ40 and Aβ42 but exhibit no overt AD-related brain pathology such as extracellular Aβ plaques or neuroinflammation [12, 18]. None of these *App*-KI mice exhibit tau pathology or severe neuron loss, suggesting that they are models of preclinical AD [11].

Recent reports demonstrated that *App*-KI mice exhibit a reduction in the number of hippocampal mushroom spines [19, 20] and disruption of neural circuit activities organized by gamma oscillations in the medial entorhinal cortex [21]. They also revealed new mechanisms underlying Aβ pathology: genetic deletion of the orphan G protein GPR3, which regulates γ-secretase activity and Aβ generation, attenuates Aβ pathology [22], whereas ablation of kallikrein-related peptidase 7 (KLK7), an astrocyte-derived Aβ-degrading enzyme, accelerates Aβ pathologies in the brains of *App*-KI mice [23]. To further understand the utility of the *App*-KI mouse models for basic and translational research, it is crucial to obtain detailed information on their behavioral phenotypes, including cognitive and non-cognitive comorbidity related to AD [7, 12, 18, 24–26].

To investigate the behavioral changes associated with Aβ pathology, we searched for alterations in cognitive and emotional domains specifically present in *App*^*NL*-*G*-*F/NL*-*G*-*F*^ mice. As our experimental paradigms, we used the elevated plus maze (EPM), contextual fear conditioning (CFC), and Barnes maze (BM) tasks. Analysis with EPM revealed that *App*^*NL*-*G*-*F/NL*-*G*-*F*^ mice exhibited robust anxiolytic-like behaviors, whereas *App*^*NL/NL*^ mice exhibited anxiogenic-like behaviors, in comparison with wild-type (WT) C57BL/6J mice. In CFC and BM, no significant learning and memory deficits were observed in *App*^*NL*-*G*-*F/NL*-*G*-*F*^ or *App*^*NL/NL*^ mice, whereas *App*^*NL*-*G*-*F/NL*-*G*-*F*^ mice exhibited a subtle decline in spatial learning ability in the BM. These results suggest that *App*^*NL*-*G*-*F/NL*-*G*-*F*^ and *App*^*NL/NL*^ mice exhibit significant changes in anxiety-related behaviors, with minimal alterations in learning ability and memory. Our results provide information about behavioral readouts in *App*-KI mice that will be useful for future basic and translational research.

## Results

In *App*^*NL*-*G*-*F/NL*-*G*-*F*^ mice, age-dependent cortical Aβ amyloidosis began by 2 months and saturated around 7 months of age (Additional file 1: Fig. S1) [12]. These mice also developed Aβ amyloidosis in the hippocampal and subcortical regions [12]. Despite aggressive Aβ amyloidosis in *App*^*NL*-*G*-*F/NL*-*G*-*F*^ mice, neuroinflammatory responses such as astrocytosis and microgliosis were not intense at the age of 6–9 months, whereas greater reactive gliosis was observed in cortical and hippocampal regions, as well as in subcortical regions, at the age of 15–18 months (Additional file 1: Fig. S1) [12, 18]. By contrast, Aβ plaques and neuroinflammatory responses were negligible even at 18 months of age in *App*^*NL/NL*^ mice, despite elevation of the Aβ level in the brain [12, 18]. Based on this neuropathological information, we carried out behavioral assays to capture cognitive (BM and CFC tasks) and emotional (EPM task) alterations in *App*-KI mice over the course of aging (Additional file 1: Fig. S1). In the experimental design, we noted that the same group of mice (Group 4) was repeatedly tested at 4, 6, and 8 months of age in the BM task (Additional file 1: Fig. S1).

### *App*^*NL*-*G*-*F/NL*-*G*-*F*^ mice exhibit anxiolytic-like behavior, whereas *App*^*NL/NL*^ mice exhibit anxiogenic-like behavior, in comparison with control WT mice

**Fig. 1:**
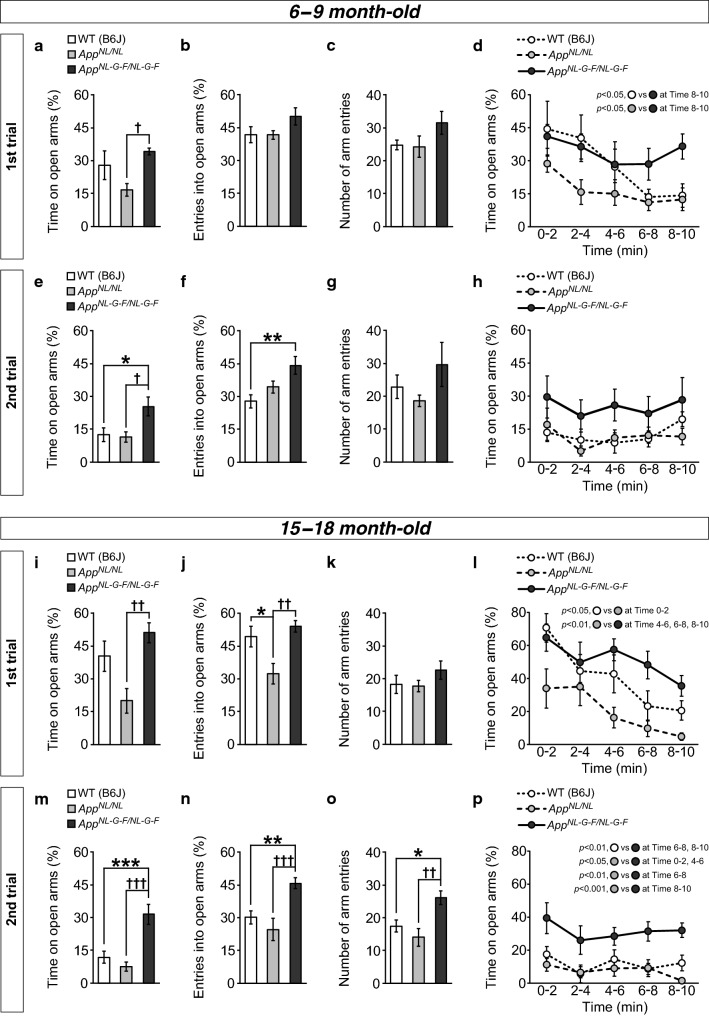
Anxiety-related behaviors in *App*^*NL*-*G*-*F/NL*-*G*-*F*^ and *App*^*NL/NL*^ mice assessed by the elevated plus maze task. Anxiety-related behaviors were assessed at both 6–9 (**a**–**h**) and 15–18 (**i**–**p**) months of age. At 6–9 months of age, *App*^*NL*-*G*-*F/NL*-*G*-*F*^ mice exhibited slightly increased levels of open arm exploration than WT mice, as indicated by the percentages of time spent on (**a**) and entries into (**b**) the open arms in the first trial. *App*^*NL/NL*^ and WT mice showed similar levels of open arm exploration. General exploratory activity in *App*^*NL*-*G*-*F/NL*-*G*-*F*^ mice was also slightly increased in comparison with WT mice (**c**). *App*^*NL*-*G*-*F/NL*-*G*-*F*^ mice persistently explored the open arms throughout the 10-min test, in contrast to the WT and *App*^*NL/NL*^ mice (**d**). In the second trial, *App*^*NL*-*G*-*F/NL*-*G*-*F*^ mice spent more time on (**e**) and entered more often into (**f**) the open arms than WT mice, with a slight increase in general activity (**g**). WT, *App*^*NL/NL*^ and *App*^*NL*-*G*-*F/NL*-*G*-*F*^ mice did not exhibit elevated avoidance responses to the open arms throughout the test in the second trial (**h**). At 15–18 months of age, *App*^*NL*-*G*-*F/NL*-*G*-*F*^ mice exhibited slight increases in open arm exploration in comparison with WT mice in the first trial, whereas *App*^*NL/NL*^ mice exhibited reduced levels of the exploration (**i** and **j**). General exploratory activity in *App*^*NL*-*G*-*F/NL*-*G*-*F*^ mice was slightly increased in comparison with WT mice (**k**). WT and *App*^*NL/NL*^ mice exhibited elevated open arm avoidance as the test progressed, whereas *App*^*NL*-*G*-*F/NL*-*G*-*F*^ mice did not (**l**). In the second trial, *App*^*NL*-*G*-*F/NL*-*G*-*F*^ mice spent more time on (**m**) and entered more often into (**n**) the open arms than WT mice. *App*^*NL*-*G*-*F/NL*-*G*-*F*^ mice also exhibited an elevation in general activity during the test (**o**). WT, *App*^*NL/NL*^ and *App*^*NL*-*G*-*F/NL*-*G*-*F*^ mice did not exhibit elevated avoidance responses to the open arms throughout the test in the second trial (**p**). 6–9 month-old; n = 8 WT (B6J), n = 8 *App*^*NL/NL*^, n = 8 *App*^*NL*-*G*-*F/NL*-*G*-*F*^. 15–18 month-old; n = 12 WT (B6J), n = 10 *App*^*NL/NL*^, n = 11 *App*^*NL*-*G*-*F/NL*-*G*-*F*^. *p < 0.05; **p < 0.01; ***p < 0.001 versus WT (B6J), ^†^*p* < 0.05; ^††^*p* < 0.01; ^†††^*p* < 0.001 versus *App*^*NL/N*L^

Anxiety-related behaviors were assessed using the EPM task, in which increased exploration of open arms indicates anxiolytic-like behavior [27, 28]. In 6–9-month-old *App*^*NL*-*G*-*F/NL*-*G*-*F*^ mice, the amount of time on (Fig. 1a; *F*[2, 21] = 4.35, *p *= 0.026, post hoc, WT vs. *App*^*NL*-*G*-*F/NL*-*G*-*F*^, *p *= 0.565) and entries into (Fig. 1b; *F*[2, 21] = 2.22, *p *= 0.133) open arms during the 10-min test were slightly increased in comparison with WT mice, although these differences were not statistically significant with our sample size. The average total number of arm entries (Fig. 1c; *F*[2, 21] = 1.95, *p *= 0.167) and the distance travelled during the 10-min test (Additional file 2: Fig. S2a and b; *F*[2, 21] = 0.27, *p *= 0.766) were also slightly increased in *App*^*NL*-*G*-*F/NL*-*G*-*F*^ mice, although these differences were not statistically significant. By contrast, *App*^*NL/NL*^ mice exhibited similar levels of the amount of time on (Fig. 1a; post hoc, WT vs. *App*^*NL/NL*^, *p *= 0.170) and entries into (Fig. 1b) open arms to those of WT mice, with no alterations in general exploratory activity (Fig. 1c, Additional file 2: Fig. S2b).

However, the patterns of exploration in *App*^*NL*-*G*-*F/NL*-*G*-*F*^ mice differed from those observed in WT mice. When we analyzed the time course of open arm exploration by scoring the percentage of time spent on the arms in each 2-min interval (Fig. 1d), WT mice exhibited significant reductions in the time spent on the open arms as the test progressed (*F*[4, 28] = 3.75, *p *= 0.014), consistent with a previous report [28–30]. By contrast, *App*^*NL*-*G*-*F/NL*-*G*-*F*^ mice persistently explored the open arms throughout the test (Fig. 1d; *F*[4, 28] = 0.68, *p *= 0.610). At a later time point, *App*^*NL*-*G*-*F/NL*-*G*-*F*^ mice spent significantly more time on the open arms than WT mice (Fig. 1d; Time 8–10, *F*[2, 21] = 6.11, *p *= 0.008, post hoc, WT vs. *App*^*NL*-*G*-*F/NL*-*G*-*F*^, *p *= 0.022). In contrast, similar to WT mice, *App*^*NL/NL*^ mice exhibited a decrease in open arm exploration as the test progressed (Fig. 1d; *F*[4, 28] = 2.69, *p *= 0.051). These results suggest that *App*^*NL*-*G*-*F/NL*-*G*-*F*^ mice have altered responses to aversive situations, such as open spaces.

Previous studies demonstrated that laboratory rodents exhibited a significant reduction of open arm exploration when re-exposed to the EPM [28, 30, 31]. This suggests that prior test experience caused a qualitative shift in emotional state, and the acquisition of a phobic state rather than an unconditioned anxiety response. To investigate whether prior test experience could alter anxiety-related behavior, we re-tested the *App*-KI and WT mice in the same EPM paradigm.

As reported previously, WT mice exhibited robust avoidance responses to the open arms in the second trial of our EPM task, reflected by reduced percentages of time spent on and entries into the arms. By contrast, 6–9-month-old *App*^*NL*-*G*-*F/NL*-*G*-*F*^ mice spent significantly more time on the open arms (Fig. 1e; *F*[2, 21] = 5.30, *p *= 0.014, post hoc, WT vs. *App*^*NL*-*G*-*F/NL*-*G*-*F*^, *p *= 0.034) and entered them more frequently (Fig. 1f; *F*[2, 21] = 6.11, *p *= 0.008, post hoc, WT vs. *App*^*NL*-*G*-*F/NL*-*G*-*F*^, *p *= 0.006) during the 10-min test period than WT mice. The time course analysis also revealed a persistent and durable exploration of open arms in *App*^*NL*-*G*-*F/NL*-*G*-*F*^ mice (Fig. 1h; *F*[4, 28] = 0.207, *p *= 0.932). These mice showed slightly higher preference toward the open arms in comparison with WT mice at each time point, although the differences were not statistically significant (Fig. 1h; Time 0–2, *F*[2, 21] = 1.33, *p *= 0.286; Time 2–4, *F*[2, 21] = 2.44, *p *= 0.111; Time 4–6, *F*[2, 21] = 3.05, *p *= 0.069; Time 6–8, *F*[2, 21] = 1.35, *p *= 0.280; Time 8–10, *F*[2, 21] = 1.59, *p *= 0.228). *App*^*NL/NL*^ and WT mice engaged in similar levels of open arm exploration (Fig. 1e, f and h). As with the case of the first trial, *App*^*NL*-*G*-*F/NL*-*G*-*F*^ mice exhibited slight increases in the average total number of arm entries (Fig. 1g; *F*[2, 21] = 1.53, *p *= 0.240) and the distance travelled during the 10-min test (Additional file 2: Fig. S2c and d; *F*[2, 21] = 1.22, *p *= 0.316), although these differences were not statistically significant with our sample size.

Taken together, these results suggest that 6–9-month-old *App*^*NL*-*G*-*F/NL*-*G*-*F*^ mice exhibit robust anxiolytic-like behavior, even after they have habituated to a test environment.

To investigate whether the observed anxiolytic-like behavior in *App*^*NL*-*G*-*F/NL*-*G*-*F*^ mice was maintained during aging, we performed the same EPM task at 15–18 months of age. In the first trial, *App*^*NL*-*G*-*F/NL*-*G*-*F*^ mice showed a tendency to spent more time on (Fig. 1i; *F*[2, 30] = 6.78, *p *= 0.004, post hoc, WT vs. *App*^*NL*-*G*-*F/NL*-*G*-*F*^, *p *= 0.401) and a slightly higher frequency of entries into (Fig. 1j; *F*[2, 30] = 7.01, *p *= 0.003, post hoc, WT vs. *App*^*NL*-*G*-*F/NL*-*G*-*F*^, *p *= 0.700) the open arms during the 10-min test than WT mice, although these differences were not statistically significant. By contrast, *App*^*NL/NL*^ mice tended to spend less time in the open arms (Fig. 1i; post hoc, WT vs. *App*^*NL/NL*^, *p *= 0.054) and entered them significantly less frequently (Fig. 1j; post hoc, WT vs. *App*^*NL/NL*^, *p *= 0.021) than WT mice, suggesting anxiogenic-like behavior in *App*^*NL/NL*^ mice. General exploratory activity was slightly increased in *App*^*NL*-*G*-*F/NL*-*G*-*F*^ mice, although the difference was not statistically significant with our sample size (Fig. 1k; *F*[2, 30] = 1.07, *p *= 0.356; Additional file 2: Fig. S2e and f; *F*[2, 30] = 0.18, *p *= 0.836).

As observed at 6–9 months of age, 15–18-month-old WT and *App*^*NL/NL*^ mice exhibited clear avoidance of the open arms as the test progressed (Fig. 1l; WT, *F*[4, 44] = 8.96, *p *< 0.001; *App*^*NL/NL*^, *F*[4, 36] = 4.15, *p *= 0.007), whereas *App*^*NL*-*G*-*F/NL*-*G*-*F*^ mice did not exhibit a significant change in open arm exploration during the test (*F*[4, 40] = 1.83, *p *= 0.141). At an early time point, *App*^*NL/NL*^ mice spent significantly less time on the open arms than WT mice (Fig. 1l; Time 0–2, *F*[2, 30] = 4.13, *p *= 0.026, post hoc, WT vs. *App*^*NL/NL*^, *p *= 0.021). By contrast, *App*^*NL*-*G*-*F/NL*-*G*-*F*^ mice exhibited slightly higher open arm exploration in the latter half of the test in comparison with WT mice, but the differences were not statistically significant (Fig. 1l; Time 4–6, *F*[2, 30] = 5.30, *p *= 0.011, post hoc, WT vs. *App*^*NL*-*G*-*F/NL*-*G*-*F*^, *p *= 0.466; Time 6–8, *F*[2, 30] = 5.74, *p *= 0.008, post hoc, WT vs. *App*^*NL*-*G*-*F/NL*-*G*-*F*^, *p *= 0.079; Time 8–10, *F*[2, 30] = 7.94, *p *= 0.002, post hoc, WT vs. *App*^*NL*-*G*-*F/NL*-*G*-*F*^, *p *= 0.125). These results suggest that 15–18-month-old *App*^*NL*-*G*-*F/NL*-*G*-*F*^ mice exhibit alterations in the habituation process to aversive stimuli.

In the second trial, 15–18-month-old *App*^*NL*-*G*-*F/NL*-*G*-*F*^ mice spent significantly more time on the open arms (Fig. 1m; *F*[2, 30] = 13.87, *p *< 0.001, post hoc, WT vs. *App*^*NL*-*G*-*F/NL*-*G*-*F*^, *p *< 0.001) and entered them more often (Fig. 1n; *F*[2, 30] = 9.37, *p *< 0.001, post hoc, WT vs. *App*^*NL*-*G*-*F/NL*-*G*-*F*^, *p *= 0.009) than WT mice. The time course analysis also revealed a persistent and durable exploration of open arms in *App*^*NL*-*G*-*F/NL*-*G*-*F*^ mice (Fig. 1p; *F*[4, 40] = 0.74, *p *= 0.570). *App*^*NL*-*G*-*F/NL*-*G*-*F*^ mice spent more time on the open arms from the beginning of the test (Fig. 1p; Time 0–2, *F*[2, 30] = 5.13, *p *= 0.012, post hoc, WT vs. *App*^*NL*-*G*-*F/NL*-*G*-*F*^, *p *= 0.053; Time 2–4, *F*[2, 30] = 3.31, *p *= 0.050; Time 4–6, *F*[2, 30] = 3.51, *p *= 0.043, post hoc, WT vs. *App*^*NL*-*G*-*F/NL*-*G*-*F*^, *p *= 0.157) and particularly at later time points (Time 6–8, *F*[2, 30] = 7.26, *p *= 0.003, post hoc, WT vs. *App*^*NL*-*G*-*F/NL*-*G*-*F*^, *p *= 0.005; Time 8–10, *F*[2, 30] = 15.14, *p *< 0.001, post hoc, WT vs. *App*^*NL*-*G*-*F/NL*-*G*-*F*^, *p *= 0.003). In addition, *App*^*NL*-*G*-*F/NL*-*G*-*F*^ mice exhibited a significant increase in the total number of arm entries in comparison with WT mice (Fig. 1o; *F*[2, 30] = 7.85, *p *= 0.002, post hoc, WT vs. *App*^*NL*-*G*-*F/NL*-*G*-*F*^, *p *= 0.021). We also measured the distance travelled during the test (Additional file 2: Fig. S2g and h) and noticed that *App*^*NL*-*G*-*F/NL*-*G*-*F*^ mice moved longer than WT mice, though the difference was not statistically significant with our sample size (*F*[2, 30] = 3.76, *p *= 0.035, post hoc, WT vs. *App*^*NL*-*G*-*F/NL*-*G*-*F*^, *p *= 0.154). In contrast to the first trial, *App*^*NL/NL*^ and WT mice exhibited similar levels of open arm exploration (Fig. 1m and n), presumably due to habituation of WT mice to the test environment.

Taken together, these results suggest that 15–18-month-old *App*^*NL*-*G*-*F/NL*-*G*-*F*^ mice exhibit robust anxiolytic-like behaviors, with increases in general exploratory activity, whereas *App*^*NL/NL*^ mice displayed unconditioned anxious phenotypes in comparison with WT mice.

### *App*^*NL*-*G*-*F/NL*-*G*-*F*^ and *App*^*NL/NL*^ mice exhibit normal learning and memory of contextual fear up to 15–18 months of age in comparison with WT mice

The CFC task is a commonly used procedure for inducing learned fear, which is believed to be hippocampal-dependent [32, 33]. In this paradigm, a particular context as a conditioned stimulus evokes fear through association with an aversive event, such as a footshock [34]. Conditioned fear responses are impaired in both human patients and mouse models of AD [35–38].

**Fig. 2:**
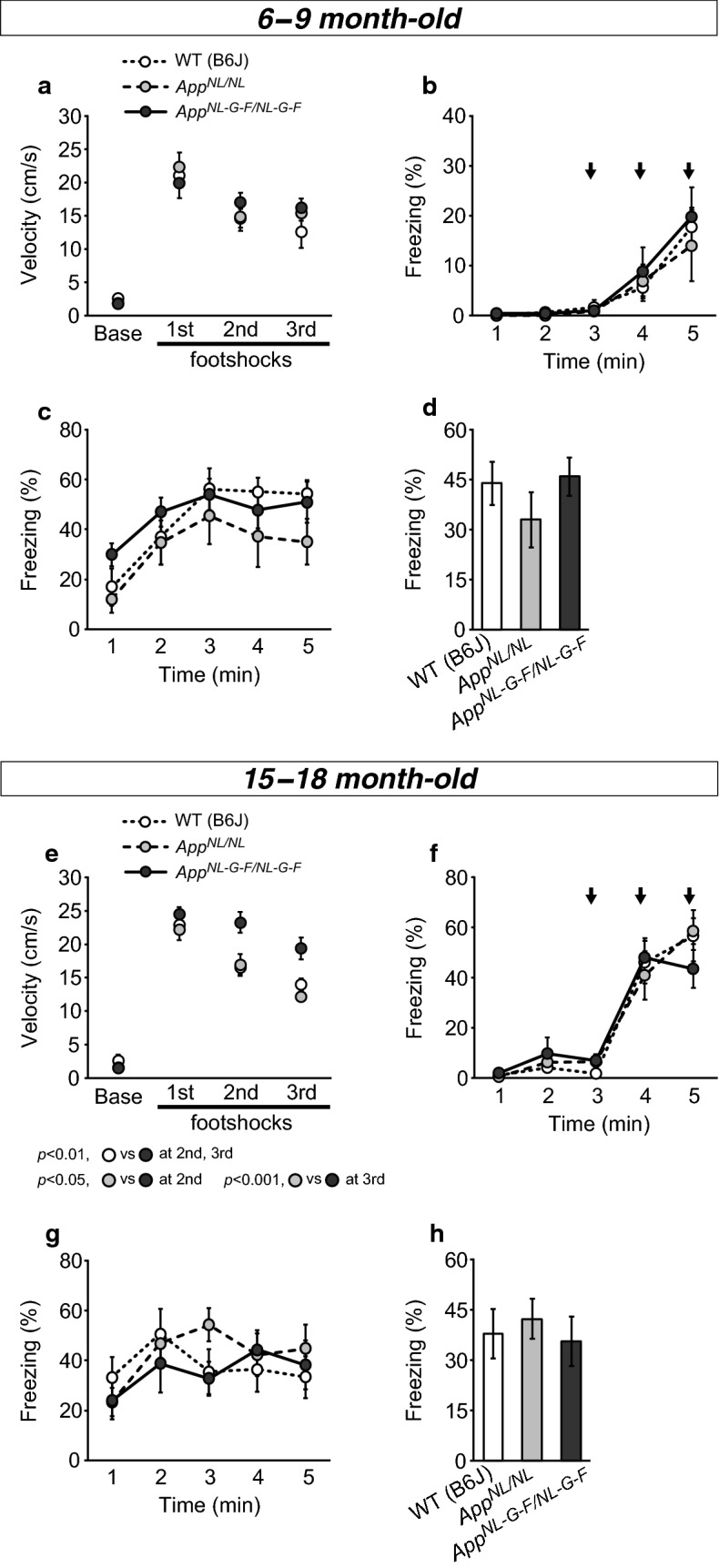
Emotional learning and fear memory in *App*^*NL*-*G*-*F/NL*-*G*-*F*^ and *App*^*NL/NL*^ mice, assessed by the contextual fear conditioning task. Learning and memory of contextual fear were assessed at both 6–9 (**a**–**d**) and 15–18 (**e**–**h**) months of age. At 6–9 months of age, *App*^*NL*-*G*-*F/NL*-*G*-*F*^ and *App*^*NL/NL*^ mice exhibited similar levels of shock reactivity, as indicated by velocity, during each presentation of footshock (**a**). During conditioning, all genotypes exhibited the same levels of freezing response to subsequent presentation of footshock (indicated by black arrows) (**b**). In the context test, all genotypes exhibited similar increases in the freezing response during the test, as revealed by the min-by-min data (**c**). Total levels of freezing response during the 5-min test period were comparable among all genotypes (**d**). At 15–18 months of age, *App*^*NL*-*G*-*F/NL*-*G*-*F*^ mice exhibited higher shock reactivity than WT mice, as revealed by an increase in velocity during the second and third footshocks (**e**). During conditioning, all genotypes exhibited the same levels of freezing response to subsequent presentation of footshock (indicated by black arrows) (**f**). The time course of the freezing response in the context test was not different among genotypes (**g**). During the 5-min test period, the percentages of time spent in the frozen state by *App*^*NL*-*G*-*F/NL*-*G*-*F*^ and *App*^*NL/NL*^ mice were similar to that in WT mice (**h**). 6–9 month-old; n = 6 WT (B6J), n = 6 *App*^*NL/NL*^, n = 9 *App*^*NL*-*G*-*F/NL*-*G*-*F*^. 15–18 month-old; n = 8 WT (B6J), n = 7 *App*^*NL/NL*^, n = 7 *App*^*NL*-*G*-*F/NL*-*G*-*F*^

At 6–9 months of age, the velocities of both *App*^*NL*-*G*-*F/NL*-*G*-*F*^ and *App*^*NL/NL*^ mice during administration of each footshock were comparable to those of WT mice (Fig. 2a; first, *F*[2, 18] = 0.32, *p *= 0.732; second, *F*[2, 18] = 0.71, *p *= 0.506; third, *F*[2, 18] = 1.30, *p *= 0.297). In addition, *App*^*NL*-*G*-*F/NL*-*G*-*F*^, *App*^*NL/NL*^, and WT mice exhibited the same levels of the freezing response upon subsequent presentation of footshocks during conditioning (Fig. 2b; genotype, *F*[2, 18] = 0.19, *p *= 0.830; time, *F*[1.6, 28.7] = 18.02, *p *< 0.001). To determine whether there were any locomotor deficits that could have confounded the outcome, we compared the distance travelled during the pre-shock period (the 3-min period prior to the first footshock) among genotypes (Additional file 3: Fig. S3a and b). At these ages, *App*^*NL*-*G*-*F/NL*-*G*-*F*^ mice seemed to be less active than WT mice during the pre-shock period, although the difference was not statistically significant with our sample size (Additional file 3: Fig. S3b; *F*[2, 18] = 2.99, *p *= 0.076). These results suggest that all genotypes were capable of detecting and responding to footshock stimuli at similar levels.

In the context test, min-by-min scoring of the percentage of freezing behavior revealed that all genotypes exhibited similar increases in the response as the test progressed (Fig. 2c; genotype, *F*[2, 18] = 10.25, *p *= 0.371; time, *F*[2.6, 46.4] = 21.08, *p *< 0.001). Moreover, levels of the freezing response during the total 5-min period were comparable among all genotypes (Fig. 2d; *F*[2, 18] = 1.05, *p *= 0.371). These results suggest that both *App*^*NL*-*G*-*F/NL*-*G*-*F*^ and *App*^*NL/NL*^ mice can learn and memorize the association between cues in the experimental chamber and footshock as effectively as WT mice.

At 15–18 months of age, *App*^*NL*-*G*-*F/NL*-*G*-*F*^ mice exhibited significantly higher shock reactivity than WT mice, as revealed by an increased velocity during the second and third footshocks (Fig. 2e; first, *F*[2, 19] = 0.85, *p *= 0.444; second, *F*[2, 19] = 7.36, *p *= 0.004, post hoc, WT vs. *App*^*NL*-*G*-*F/NL*-*G*-*F*^, *p *= 0.007; third, *F*[2, 19] = 10.82, *p *< 0.001, post hoc, WT vs. *App*^*NL*-*G*-*F/NL*-*G*-*F*^, *p *= 0.008). This result suggests that 15–18-month-old *App*^*NL*-*G*-*F/NL*-*G*-*F*^ mice have heightened sensitivity to painful stimuli. During conditioning, both *App*^*NL*-*G*-*F/NL*-*G*-*F*^ and *App*^*NL/NL*^ mice exhibited levels of freezing upon subsequent presentation of footshocks similar to those of WT mice (Fig. 2f; genotype, *F*[2, 19] = 0.0, *p *= 0.994; time, *F*[1.9, 36.5] = 84.15, *p *< 0.001). We also found that *App*^*NL/NL*^ mice moved significantly less than WT mice during the pre-shock period (Additional file 3: Fig. S3c and d; *F*[2, 19] = 5.13, *p *= 0.017, post hoc, WT vs. *App*^*NL/NL*^, *p *= 0.016). However, a slight reduction in locomotor activity in *App*^*NL/NL*^ mice does not significantly affect the behavioral outcomes of the CFC task in *App*^*NL/NL*^ mice, since these mice can exhibit similar levels of shock reactivity and freezing behavior with WT mice (Fig. 2e and f). Locomotor activity during the pre-shock period was also slightly decreased in *App*^*NL*-*G*-*F/NL*-*G*-*F*^ mice, but the difference was not statistically significant with our sample size (Additional file 3: Fig. S3d; post hoc, WT vs. *App*^*NL*-*G*-*F/NL*-*G*-*F*^, *p *= 0.092).

In the context test, the min-by-min data for freezing behavior revealed that the time course of the freezing response was similar among all genotypes (Fig. 2g; genotype, *F*[2, 19] = 0.23, *p *= 0.799; time, *F*[4, 76] = 5.06, *p *= 0.001). During the total 5-min period of the test, both *App*^*NL*-*G*-*F/NL*-*G*-*F*^ and *App*^*NL/NL*^ mice exhibited levels of freezing behavior similar to those of WT mice (Fig. 2h; *F*[2, 19] = 0.23, *p *= 0.800).

Taken together, these results suggest that both *App*^*NL*-*G*-*F/NL*-*G*-*F*^ and *App*^*NL/NL*^ mice have intact learning and memory of contextual fear, even at 15–18 months of age.

### *App*^*NL*-*G*-*F/NL*-*G*-*F*^ mice exhibit alterations in spatial learning ability, with intact memory, in the BM task at 8 months of age

**Fig. 3:**
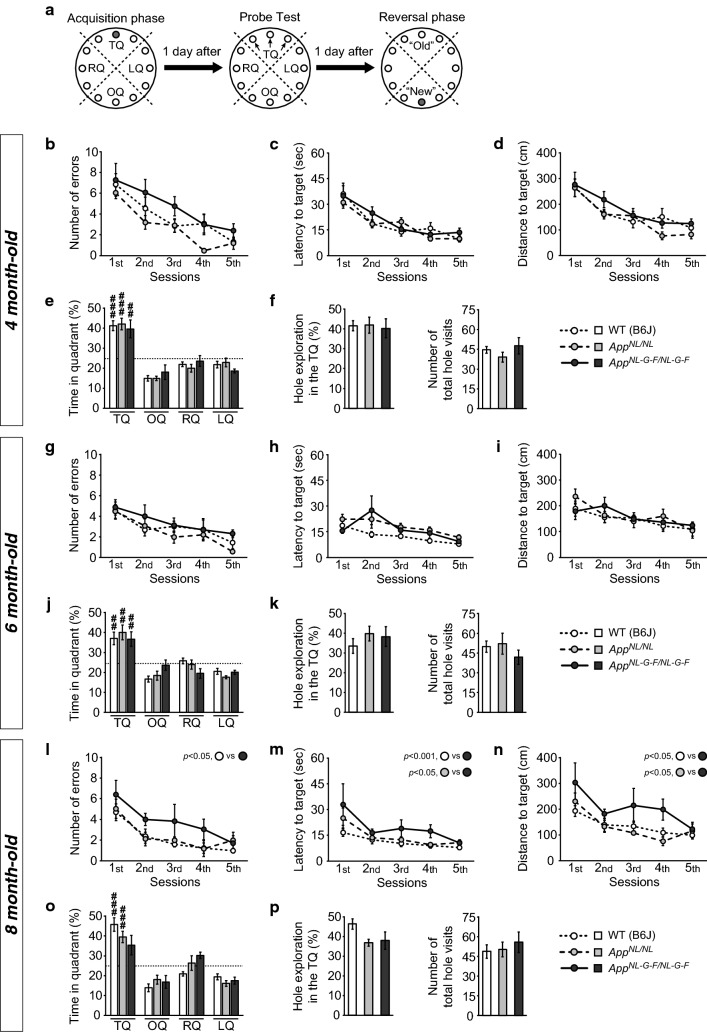
Spatial learning and memory in *App*^*NL*-*G*-*F/NL*-*G*-*F*^ and *App*^*NL/NL*^ mice assessed by the Barnes maze task. Spatial learning and memory were assessed at 4 (**b**–**f**), 6 (**g**–**k**), and 8 (**i**–**p**) months of age. In the acquisition phase (**a** [left]), one hole (indicated by a gray hole) was designated as the target hole with an escape box. A probe test was performed 1 day after the last training session, in which the escape box was removed (**a** [middle]). The three black arrows indicate the target hole and adjacent holes, respectively. In the reversal phase (**a** [right]), the target hole was moved to the position opposite the original 1 day after the probe test. TQ: target quadrant; OQ: opposite quadrant; RQ: right quadrant; LQ: left quadrant. At 4 and 6 months of age, *App*^*NL*-*G*-*F/NL*-*G*-*F*^ and *App*^*NL/NL*^ mice performed as well as WT mice in acquisition of the target hole, as revealed by similar decreases in the number of errors (**b** and **g**), latency (**c** and **h**), and distance (**d** and **i**) across the acquisition phase. In the probe test, all genotypes exhibited similar levels of preference toward the target quadrant (TQ) above chance level (25%, as indicated by dotted lines) (**e** and **j**) and similar levels of exploration of the holes in the target quadrant (**f** [left] and **k** [left]) with no differences in exploratory activity (**f** [right] and **k** [right]). At 8 months of age, *App*^*NL*-*G*-*F/NL*-*G*-*F*^ mice made more errors (**l**), took more time (**m**), and travelled farther (**n**) to reach the target hole than WT mice throughout the acquisition phase. In the probe test, WT and *App*^*NL/NL*^ mice exhibited significant preference toward the target quadrant (TQ) above chance level (25%, as indicated by dotted lines) (**o**). All genotypes exhibited similar levels of exploration of the holes in the target quadrant (**p** [left]), with no alterations in general activity (**p** [right]). 4 month-old; n = 8 WT (B6J), n = 8 *App*^*NL/NL*^, n = 7 *App*^*NL*-*G*-*F/NL*-*G*-*F*^. 6 month-old; n = 8 WT (B6J), n = 8 *App*^*NL/NL*^, n = 7 *App*^*NL*-*G*-*F/NL*-*G*-*F*^. 8 month-old; n = 8 WT (B6J), n = 7 *App*^*NL/NL*^, n = 6 *App*^*NL*-*G*-*F/NL*-*G*-*F*^. ^♯♯^*p* < 0.01, ^♯♯♯^*p* < 0.001 versus chance level

The BM task is a spatial memory task that requires animals to learn the location of an escape hole using spatial cues, and is therefore thought to be hippocampal-dependent [39, 40]. This task is commonly used for assessment of memory deficits in animal models of AD [41–43]. In our experiments, mice were asked to acquire the spatial location of a target hole that was connected to a dark escape box during the acquisition phase (Fig. 3a [left]). One day after the fifth session of the acquisition phase, a probe test was conducted without an escape box to investigate whether mice had learned the location of the target hole by extra-maze cues (Fig. 3a [middle]). To further assess cognitive flexibility, mice were subjected to the reversal learning task (five sessions) 1 day after the probe test (Fig. 3a [right]). And as mentioned in the experimental design above, the same group of mice was repeatedly tested at 4, 6, and 8 months of age in this BM task (Additional file 1: Fig. S1).

We found that *App*^*NL/NL*^, *App*^*NL*-*G*-*F/NL*-*G*-*F*^, and WT mice performed equally well in acquisition of the target hole in the BM at the ages of 4 months (Fig. 3b; *F*[2, 20] = 3.12, *p *= 0.066, Fig. 3c; *F*[2, 20] = 0.48, *p *= 0.625, Fig. 3d; *F*[2, 20] = 1.10, *p *= 0.353) and 6 months (Fig. 3g; *F*[2, 20] = 1.27, *p *= 0.303, Fig. 3h; *F*[2, 20] = 2.80, *p *= 0.085, Fig. 3i; *F*[2, 20] = 0.24, *p *= 0.788). The number of errors (Fig. 3b; *F*[4, 80] = 23.21, *p *< 0.001, Fig. 3g; *F*[2.7, 54.8] = 10.07, *p *< 0.001), latency (Fig. 3c; *F*[1.9, 37.6] = 29.26, *p *< 0.001, Fig. 3h; *F*[1.7, 33.1] = 8.06, *p *= 0.002), and distance (Fig. 3d; *F*[2.3, 45.3] = 23.18, *p *< 0.001, Fig. 3i; *F*[2.8, 56.5] = 7.11, *p *= 0.001) to reach the target hole significantly decreased as the session progressed, suggesting that all genotypes had similar learning ability.

In the probe test, all genotypes exhibited similar levels of preference toward the target quadrant that contained the target hole and the two adjacent holes at both 4 months (Fig. 3e; genotype, *F*[2, 20] = 1.06, *p *= 0.365; quadrant, *F*[2.0, 40.3] = 49.06, *p *< 0.001) and 6 months of age (Fig. 3j; genotype, *F*[2, 20] = 1.56, *p *= 0.235; quadrant, *F*[1.6, 32.0] = 30.84, *p *< 0.001). A percentage of time spent in the target quadrant for each genotype was significantly higher than chance level (25%) at both 4 months (Fig. 3e; WT, *t*(14) = 6.59, *p *< 0.001; *App*^*NL/NL*^, *t*(14) = 5.88, *p *< 0.001; *App*^*NL*-*G*-*F/NL*-*G*-*F*^, *t*(12) = 3.28, *p *= 0.007) and 6 months of age (Fig. 3j; WT, *t*(14) = 3.91, *p *= 0.002; *App*^*NL/NL*^, *t*(14) = 4.07, *p *= 0.001; *App*^*NL*-*G*-*F/NL*-*G*-*F*^, *t*(12) = 3.22, *p *= 0.007). Moreover, all genotypes exhibited similar levels of exploration of the holes in the target quadrant, with no differences in general exploratory activity, at both 4 months (Fig. 3f; (left) *F*[2, 20] = 0.05, *p *= 0.949; (right) *F*[2, 20] = 1.03, *p *= 0.375) and 6 months of age (Fig. 3k; (left) *F*[2, 20] = 0.67, *p *= 0.524; (right) *F*[2, 20] = 0.75, *p *= 0.487). These results suggest that both *App*^*NL*-*G*-*F/NL*-*G*-*F*^ and *App*^*NL/NL*^ mice had intact spatial learning and memory at 4 and 6 months of age.

At 8 months of age, *App*^*NL*-*G*-*F/NL*-*G*-*F*^ mice exhibited a significant increase in the number of errors (Fig. 3l; *F*[2, 18] = 5.34, *p *= 0.015, post hoc, WT vs. *App*^*NL*-*G*-*F/NL*-*G*-*F*^, *p *= 0.015), latency (Fig. 3m; *F*[2, 18] = 10.28, *p *= 0.001, post hoc, WT vs. *App*^*NL*-*G*-*F/NL*-*G*-*F*^, *p *< 0.001), and distance (Fig. 3n; *F*[2, 18] = 6.24, *p *= 0.009, post hoc, WT vs. *App*^*NL*-*G*-*F/NL*-*G*-*F*^, *p *= 0.016) in comparison with WT mice. However, *App*^*NL*-*G*-*F/NL*-*G*-*F*^ mice still exhibited a significant decrease in the number of errors (Fig. 3l; *F*[2.5, 45.2] = 11.47, *p *< 0.001), latency (Fig. 3m; *F*[1.4, 25.2] = 6.91, *p *= 0.008), and distance (Fig. 3n; *F*[2.1, 38.1] = 8.35, *p *= 0.001), and were able to solve the task proficiently (at levels comparable to those of WT mice) by the fifth training session. These results suggest that 8-month-old *App*^*NL*-*G*-*F/NL*-*G*-*F*^ mice have subtle alterations in their ability to learn the spatial location of the target hole.

In the probe test, all genotypes exhibited similar levels of preference toward the target quadrant (Fig. 3o; genotype, *F*[2, 18] = 1.36, *p *= 0.283; quadrant, *F*[1.8, 31.9] = 38.63, *p *< 0.001). The percentages of time spent in the target quadrant were significantly higher above chance level for WT and *App*^*NL/NL*^ mice (Fig. 3o; WT, *t*(14) = 6.00, *p *< 0.001; *App*^*NL/NL*^, *t*(12) = 5.07, *p *< 0.001), but not for *App*^*NL*-*G*-*F/NL*-*G*-*F*^ mice (*t*(10) = 2.11, *p *= 0.062), with our sample size. The percentage of hole exploration in the target quadrant (Fig. 3p; (left) *F*[2, 18] = 3.35, *p *= 0.058) and the total number of hole visits (Fig. 3p; (right) *F*[2, 18] = 0.35, *p *= 0.712) were similar among all genotypes.

Taken together, these results suggest that, at 8 months of age, *App*^*NL*-*G*-*F/NL*-*G*-*F*^ mice exhibit reduced spatial learning ability in comparison with WT mice, but still retain normal spatial memory.

### Both *App*^*NL*-*G*-*F/NL*-*G*-*F*^ and *App*^*NL/NL*^ mice exhibit normal flexibility in a reversal learning task up to 8 months of age

Reversal learning, a way to model some aspects of higher-order cognitive functions in rodents [44, 45], requires cognitive flexibility and impulse control, and thus taps into components of human executive function [46, 47]. Previous studies demonstrated that transgenic mouse models of Aβ amyloidosis are impaired in reversal learning [48–50]. To assess reversal learning using the BM, we moved the target hole to the opposite position 1 day after the probe test (Fig. 3a [right]).

**Fig. 4:**
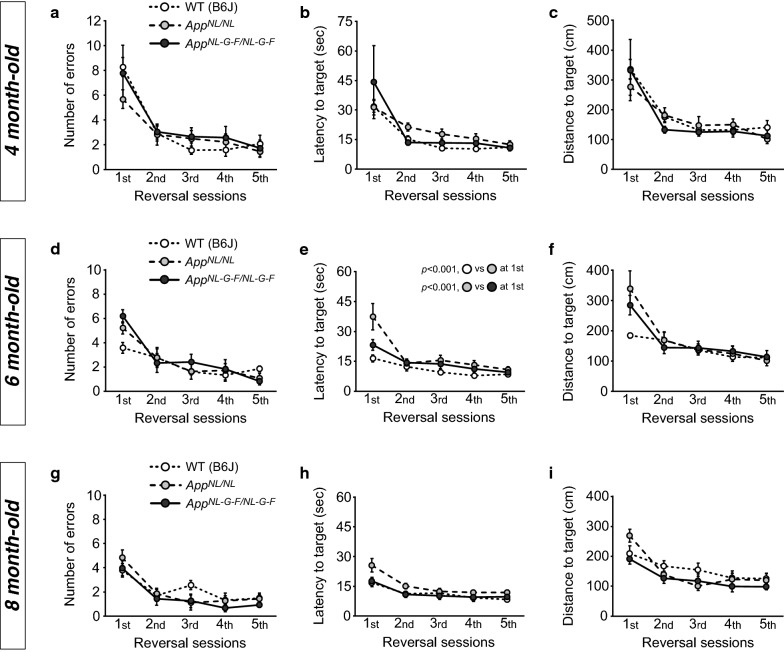
Behavioral flexibility in *App*^*NL*-*G*-*F/NL*-*G*-*F*^ and *App*^*NL/NL*^ mice assessed by spatial reversal leaning task using the Barnes maze. Behavioral flexibility was assessed at 4 (**a**–**c**), 6 (**d**–**f**), and 8 (**g**–**i**) months of age by spatial reversal learning task using the Barnes maze. At all ages, *App*^*NL*-*G*-*F/NL*-*G*-*F*^ and *App*^*NL/NL*^ mice performed equally well in acquisition of the new target hole in comparison with WT mice, as indicated by decreases in the number of errors (**a**, **d**, and **g**), latency (**b**, **e**, and **h**), and distance (**c**, **f**, and **i**) across the reversal sessions. At 6 months of age, *App*^*NL/NL*^ mice took more time to reach the new target hole only in the first session of the reversal phase (**e**). 4 month-old; n = 8 WT (B6J), n = 8 *App*^*NL/NL*^, n = 7 *App*^*NL*-*G*-*F/NL*-*G*-*F*^. 6 month-old; n = 8 WT (B6J), n = 8 *App*^*NL/NL*^, n = 7 *App*^*NL*-*G*-*F/NL*-*G*-*F*^. 8 month-old; n = 8 WT (B6J), n = 7 *App*^*NL/NL*^, n = 6 *App*^*NL*-*G*-*F/NL*-*G*-*F*^

We found that both *App*^*NL*-*G*-*F/NL*-*G*-*F*^ and *App*^*NL/NL*^ mice exhibited similar levels of performance in the reversal learning task in comparison with WT mice at 4 months (Fig. 4a; *F*[2, 20] = 0.35, *p *= 0.711, Fig. 4b; *F*[2, 20] = 0.87, *p *= 0.434, Fig. 4c; *F*[2, 20] = 0.32, *p *= 0.733), 6 months (Fig. 4d; *F*[2, 20] = 0.38, *p *= 0.690, Fig. 4e; *F*[2, 20] = 6.31, *p *= 0.008, Fig. 4f; *F*[2, 20] = 1.73, *p *= 0.202), and 8 months of age (Fig. 4g; *F*[2, 18] = 0.66, *p *= 0.530, Fig. 4h; *F*[2, 18] = 3.00, *p *= 0.075, Fig. 4i; *F*[2, 18] = 1.41, *p *= 0.269).

The number of errors (Fig. 4a; *F*[1.8, 36.4] = 28.55, *p *< 0.001, Fig. 4d; *F*[2.5, 49.8] = 30.91, *p *< 0.001, Fig. 4g; *F*[2.2, 40.0] = 28.20, *p *< 0.001), latency (Fig. 4b; *F*[1.1, 22.9] = 13.28, *p *= 0.001, Fig. 4e; *F*[1.3, 25.6] = 24.78, *p *< 0.001, Fig. 4h; *F*[2.1, 38.6] = 29.80, *p *< 0.001) and distance (Fig. 4c; *F*[1.5, 29.1] = 20.47, *p *< 0.001, Fig. 4f; *F*[1.4, 28.6] = 21.32, *p *< 0.001, Fig. 4i; *F*[2.4, 42.7] = 21.93, *p *< 0.001) to the new target hole were progressively reduced in all genotypes and at all ages, suggesting that both *App*^*NL*-*G*-*F/NL*-*G*-*F*^ and *App*^*NL/NL*^ mice could adjust their response to find the new location as effectively as WT mice.

We noticed that 6-month-old *App*^*NL/NL*^ mice spent more time to reach the new target hole than WT mice at the first session of the reversal phase (Fig. 4e; genotype × session, *F*[2.6, 25.6] = 3.47, *p *= 0.037, simple main effect on first session, *p *< 0.001, post hoc, WT vs. *App*^*NL/NL*^, *p *< 0.001). However, no significant change in the latency was detected at the second session (simple main effect on second session, *p *= 0.833). Moreover, no such difference was observed in the 8-month-old *App*^*NL/NL*^ mice (Fig. 4h).

Taken together, these results suggest that both *App*^*NL*-*G*-*F/NL*-*G*-*F*^ and *App*^*NL/NL*^ mice exhibit normal cognitive flexibility in the reversal learning task up to 8 months of age.

## Discussion

Aβ amyloidosis, tau aggregation, neuroinflammation, neurodegeneration, and cognitive deficits are defining features of AD. To date, multiple transgenic mouse models overexpressing human APP with familial AD mutations have been shown to develop age-dependent Aβ amyloidosis, neuroinflammation, and cognitive impairments in spatial memory and contextual fear memory [6, 7, 51]. However, these APP-overexpressing mouse models may have pathophysiological properties caused by non-physiological overexpression of APP, in addition to Aβ pathology [11].

To overcome this issue, several *App*-KI mouse models have been generated that recapitulate Aβ pathology without APP overexpression. The first reported *App*-KI line, *App*^*NLh*^ mice, harbor the Swedish mutation with humanized Aβ sequences in the murine *App* gene locus. Although *App*^*NLh*^ mice do not develop Aβ pathology, the double KI line interbred with mutant *Psen1*^*P264L/P264L*^ KI line (*App*^*NLh/NLh*^ × *Psen1*^*P264L/P264L*^ mice) progressively develop Aβ pathology and cognitive impairments with no alterations in locomotor activity and anxiety-related behavior [52, 53]. More recently developed *App*^*DSL*^ mice harbor three familial AD-associated mutations (Swedish, London, and Dutch) with a humanized Aβ sequence in the murine *App* locus [54]. Similar to the *App*^*NLh*^ mice, *App*^*DSL*^ mice do not develop Aβ pathology independently, but do so when interbred with *Psen1*^*M146V*^ KI mice. The double KI line *App*^*DSL/DSL*^ × *Psen1*^*M146V/M146V*^ also exhibits elevated levels of anxiety, followed by deficits in spatial learning and memory [54]. However, these earlier *App*-KI mouse models required homozygous expression of familial AD mutant *Psen1* alleles to obtain Aβ deposition [52, 54, 55], and the potential effects of homozygous mutation in *Psen1* on observed phenotypes must be considered.

By contrast, *App*^*NL*-*G*-*F/NL*-*G*-*F*^ mice exhibit progressive amyloid pathology, including microglial and astrocytic activation and loss of synaptic markers, in the absence of *Psen1* mutation [12, 18]. Reports of cognitive deficits in *App*^*NL*-*G*-*F/NL*-*G*-*F*^ mice have varied between laboratories [12, 25, 26], although mild impairment in spatial reversal learning and enhanced impulsivity have been detected using an automated IntelliCage apparatus [18]. These results suggest that, despite aggressive Aβ pathology and neuroinflammation, cognitive alterations in *App*^*NL*-*G*-*F/NL*-*G*-*F*^ mice are modest.

Consistent with these previous studies, our results demonstrated that neither *App*^*NL*-*G*-*F/NL*-*G*-*F*^ nor *App*^*NL/NL*^ mice exhibited severe memory deficits in the CFC (Fig. 2) or BM tasks (Fig. 3). However, we detected a subtle decline in spatial learning ability in *App*^*NL*-*G*-*F/NL*-*G*-*F*^ mice at the age of 8 months (Fig. 3). Because the learning deficits were not evident at younger ages (4 and 6 months of age), this may represent an aspect of age-dependent cognitive impairment in *App*^*NL*-*G*-*F/NL*-*G*-*F*^ mice. Moreover, alterations in acquisition of spatial information may occur before the onset of memory deficit in *App*^*NL*-*G*-*F/NL*-*G*-*F*^ mice. In our experimental design, the BM task was not run at a similar age range to the EPM and CFC tasks (Additional file 1: Fig. S1). To clarify whether *App*^*NL*-*G*-*F/NL*-*G*-*F*^ mice show more dramatic deficits in spatial learning and memory during aging, it would be important to examine a spatial task after 8 months of age.

Several previous studies have shown that commonly used transgenic models such as the Tg2576 and *App*^*Swe*^/*Psen1*^*dE9*^ mice exhibit deficits in spatial learning in the BM [41–43]. Of particular interest, the TgCRND8 mouse model exhibit poor spatial learning in the BM task [56], while they also have deficits in attentional control [57]. Given that attentional deficits are likely to influence performance on memory tasks, it is conceivable that reduced attentional control of *App*^*NL*-*G*-*F/NL*-*G*-*F*^ mice [18] might have contributed to alterations in spatial learning ability in our BM task.

In this study, we repeatedly subjected the same group of mice to the BM task until the age of 8 months (Additional file 1: Fig. S1), suggesting that mice might become familiar with the rules and environment of the maze. In fact, habituation of the testing environment and rule learning by repeated exposure to the maze resulted in the fewer errors at the age of 8 months in comparison with younger ages (Figs. 3 and 4). Thus, as suggested by a previous study in *App*^*Swe*^/*Psen1*^*dE9*^ mice using the BM task [42], these processes might be compromised in 8-month-old *App*^*NL*-*G*-*F/NL*-*G*-*F*^ mice, which may lead to observed spatial learning impairment. These results also suggest that the experimental strategy testing *App*-KI mouse models in the BM task without prior test experience is likely to yield different results observed in this study.

In addition to cognitive deficits, 60–80% of AD cases are associated with non-cognitive neuropsychiatric symptoms [58, 59], including anxiety disturbances, depressive symptoms, activity disturbances, and aggression [60–62]. For example, some AD patients are subjected to anxiety, whereas the opposite tendency (disinhibition) has also been reported [60, 61]. Intriguingly, several APP-overexpressing mouse models exhibit anxiety disturbances [7, 24] and an increase in open arm exploration has been observed in several APP-overexpressing mouse models with parenchymal Aβ plaques, including APP23, Tg2576, and *App*^*Swe*^/*Psen1*^*dE9*^ mice [24].

A very recent study reported anxiolytic-like behavior in *App*^*NL*-*G*-*F/NL*-*G*-*F*^ mice in comparison with *App*^*NL/NL*^ mice, detectable from 3 months of age [25]. Similarly, we found that *App*^*NL*-*G*-*F/NL*-*G*-*F*^ mice exhibited anxiolytic-like behavior in comparison with WT mice (Fig. 1). These data from *App*-KI mice suggest that anxiolytic-like behaviors observed in mouse models of Aβ amyloidosis are associated with Aβ-mediated pathologies [24, 53] rather than overexpression of APP. Interestingly, some mouse models with traumatic brain injury exhibit increases in open arm exploration in the EPM task, followed by elevated levels of reactive gliosis and cerebrovascular dysfunction [63–66]. Another study demonstrated that local neuroinflammation within the dorsal raphe nucleus, resulting in serotonergic hypofunction, caused the same behavioral consequences in the EPM task [67]. Because elevated levels of reactive gliosis are associated with Aβ pathology in *App*^*NL*-*G*-*F/NL*-*G*-*F*^ mice [12], these pathological changes (including neuroinflammatory responses and vascular dysfunction) may play a role in the expression of anxiolytic-like behavior.

We also noticed that activity of *App*^*NL*-*G*-*F/NL*-*G*-*F*^ mice was slightly higher than that of WT mice in the EPM task (Fig. 1 and Additional file 2: Fig. S2), raising a possibility that there may be a general increase in locomotor activity in *App*^*NL*-*G*-*F/NL*-*G*-*F*^ mice. However, in the CFC task, *App*^*NL*-*G*-*F/NL*-*G*-*F*^ mice were rather less active than WT mice during the pre-shock period (Additional file 3: Fig. S3). These results suggest that the hyperactive phenotypes observed in the EPM task may be elicited by an aversive situation, rather than an innate behavioral trait in *App*^*NL*-*G*-*F/NL*-*G*-F^ mice.

We also found that *App*^*NL/NL*^ mice, which do not develop Aβ pathology, exhibited lower open arm durations at 15–18 months of age, suggesting elevated anxiety levels in these mice. A previous study also reported that transgenic mice overexpressing APP^Swe^ without Aβ pathology exhibited elevated anxiety levels in the same EPM paradigm [24, 68]. Increased anxiety levels in these mice are not due to overproduction of N- or C-terminal fragment-β (NTF-β or CTF-β) of APP, as demonstrated by the observation that *App*^*NL*-*G*-*F/NL*-*G*-*F*^ mice exhibited anxiolytic-like behavior, whereas Tg13592 mice overexpressing CTF-β did not exhibit altered anxiety levels in comparison with their non-transgenic controls [69]. Although *App*^*NL/NL*^ mice develop neither Aβ pathology nor neuroinflammatory response, they have dramatically increased levels of soluble Aβ in comparison with WT mice. Thus, higher levels of soluble Aβ may induce changes in synaptic functions, which may be responsible for emotional control in these KI mice. Hyperanxious behavior in the EPM task is often associated with alterations in several neurotransmitter systems, including γ-aminobutyric acid (GABA)ergic and serotonergic neurotransmission [70, 71]. Thus, altered neurotransmission caused by high levels of soluble Aβ may contribute to the expression of anxiogenic-like behavior in *App*^*NL/NL*^ mice.

## Conclusion

Our results demonstrate that *App*^*NL*-*G*-*F/NL*-*G*-*F*^ and *App*^*NL/NL*^ mice exhibit behavioral changes associated with non-cognitive, emotional domains before the onset of definitive cognitive deficits. These observations consistently indicate that *App*^*NL*-*G*-*F/NL*-*G*-*F*^ mice represent a model for preclinical AD and that they are useful for the study of AD prevention rather than treatment after neurodegeneration. This study provides information that will be critical for both translational and basic research for AD using *App*^*NL*-*G*-*F/NL*-*G*-*F*^ and *App*^*NL/NL*^ mice.

## Methods

### Animals

The original lines of *App*-KI (*App*^*NL*-*G*-*F/NL*-*G*-*F*^ and *App*^*NL/NL*^) mice were established as C57BL/6J congenic line (a genetic background strain) by repeated backcrosses as described previously [12] and obtained from RIKEN Center for Brain Science (Wako, Japan). All experiments were performed with male *App*^*NL*-*G*-*F/NL*-*G*-*F*^, *App*^*NL/NL*^ and WT (C57BL/6J) mice at the Institute for Animal Experimentation in National Center for Geriatrics and Gerontology. After weaning at postnatal day (PND) 28–35, all mice were housed socially in same-sex groups in a temperature-controlled environment under a 12-h light/dark cycle (lights on at 7:00, lights off at 19:00), with food and water available ad libitum. We prepared four independent groups of male mice with mixed genotypes (*App*^*NL*-*G*-*F/NL*-*G*-*F*^, *App*^*NL/NL*^, and WT) and assessed cognitive and emotional domains using three behavioral paradigms at different ages (Additional file 1: Fig. S1). All handling and experimental procedures were performed in accordance with the Guidelines for the Care of Laboratory Animals of National Center for Geriatrics and Gerontology (Obu, Japan). All animals were euthanized with intraperitoneal barbiturate overdose (sodium pentobarbital, 120 mg/kg body weight) after each behavioral experiment.

### Elevated plus maze task

The apparatus consisted of two opposing open arms (25 × 5 cm) and two opposing closed arms (25 × 5 cm, surrounded by 15 cm-high transparent walls) that extended from a center platform (5 × 5 cm) forming a cross shape (O’hara & Co., Tokyo, Japan) [72]. The maze was elevated 50 cm above the floor with a light intensity on the center platform of approximately 100 lx. To avoid falls, the open arms were surrounded by a 0.3 cm-high rim. On the test day, 6–9-(*n *= 8/genotype) and 15–18-(*n *= 10–12/genotype) month-old mice were placed individually in the center platform facing an open arm and allowed to freely explore the apparatus for 10 min. At the end of the first trial, the mice were returned to their homecage and socially housed until the beginning of the second trial. The apparatus was cleaned with distilled water and then ethanol to remove any odor cues between subject mice. All animals were retested after 10–27 days from the first trial. Time spent on open arms (s), total distance travelled (cm) and numbers of open and closed arm entries were automatically measured by the ANY-Maze video tracking software (Stoelting Co., IL, USA) [73]. The number of open and closed arm entries was combined to yield a measure of total arm entries, which reflected general exploratory activity during the test. Open arm entries were analyzed as a percentage score by dividing the number of open arm entries by the total number of arm entries (% Entries into open arms = [Number of open arm entries/Number of total arm entries] × 100).

### Contextual fear conditioning task

Mice were handled for 3 days prior to the commencement of contextual fear conditioning. The mice were trained and tested in conditioning chambers (17 × 10 × 10 cm) with a stainless-steel grid floor (0.2 cm diameter, spaced 0.5 cm apart; O’hara & Co., Tokyo, Japan) [72] surrounded by a sound-attenuating white chest (approximately 200 lx in the chest) with a background noise (55 dB). On the conditioning day, 6–9-(*n *= 6–9/genotype) and 15–18-(*n *= 7–8/genotype) month-old mice were individually placed in the conditioning chamber and allowed to explore freely for 3 min. At the end of this 3-min period, a mild footshock (0.5 mA, 2 s) was presented. Two more footshocks were presented with a 1-min inter-stimulus interval, and then the mice were returned to their home cage at 30 s after the last footshock. One day after the conditioning, mice were placed in the same chamber and allowed to explore freely for 5 min without footshock administration. The chambers were cleaned with distilled water and then ethanol to remove any odor cues between subject mice. In each test, percentage of freezing time and distance travelled (cm) were calculated automatically using ImageFZ software (O’hara & Co., Tokyo, Japan). To assess sensitivity or reactivity to footshock, we also calculated the velocity during each footshock presentation and an equivalent baseline period (actual 2-s period just prior to the first footshock), based on distance travelled during a given time period, since it has been suggested to be the most sensitive aspect of shock reactivity [34, 74, 75].

### Barnes maze task

The Barnes circular maze task was conducted on a white circular surface (1.0 m in diameter, with 12 holes equally spaced around the perimeter; O’hara & Co., Tokyo, Japan) [72]. The circular open field was elevated 75 cm above the floor with a light intensity on the center of the circular open field of approximately 1000 lx. A black acrylic escape box (17 × 13 × 7 cm) was located under one of the holes, and the hole above the escape box represented the target hole. The location of the target hole was consistent for a given mouse, and mice within a squad were assigned to the same target hole location across the sessions. Trials were administered in a spaced fashion so that all mice within a squad completed a given trial before subsequent trials were run. The maze was rotated 90° between trials, with the spatial location of the target hole unchanged with respect to the distal visual room cues, to prevent a bias based on olfactory or proximal cues within the maze. After each trial, the apparatus including the cylinder and escape box were cleaned carefully with distilled water and then ethanol to eliminate any potential odor cues.

One day after the habituation to familiarize mice with the maze and the escape box, they were subjected to 5 days training sessions (four trials per session). The same group of mice was repeatedly tested at 4, 6 and 8 months of age (Additional file 1: Fig. S1). During the acquisition phase, 4-(*n *= 7–8/genotype), 6-(*n *= 7–8/genotype) and 8-(*n *= 6–8/genotype) month-old mice were individually placed in a white acrylic cylinder (17 cm-high, 11 cm diameter) before the start of each trial, and after approximately 30 s the cylinder was removed to start the trial. Each trial ended when the mouse entered the escape box or after 5 min had elapsed. The mice that could not find the target hole were guided to the hole manually and allowed to enter the escape box to remain there for 1 min. For each trial, the number of errors, latency (s) and distance travelled (cm) to reach the target hole were automatically measured by custom-written software in MATLAB (The MathWorks, Inc., MA, USA). In our software, target zones were defined to include each separate hole and 1 cm around them. We recorded an error when a mouse touched a target zone that did not have an escape box beneath it.

One day after the last training session, a probe test was conducted without the escape box for 3 min, to confirm that this spatial task was acquired based on navigation by distal environmental cues. The time spent in each quadrant (TQ; target quadrant, OQ; opposite quadrant, RQ; right quadrant, LQ; left quadrant) (s) and numbers of the visits to the target hole and two adjacent holes (indicated by black arrows in Fig. 3a) and total hole visits during the test were measured by the software. Hole exploration in the target quadrant was defined by percentage of the visits to three holes in the target quadrant for total hole visits during the test.

For reversal leaning task (reversal phase), the target was moved to a new position opposite to the original 1 day after the probe test, and mice were retrained in 5 days reversal sessions to find the new location of the escape box. During the reversal phase, the number of errors, latency (s), and distance travelled (cm) to reach the new target were also calculated by the software.

### Statistical analysis

Statistical differences between genotypes against behavioral parameters with one dependent variable were determined by repeated-measures analysis of variance (ANOVA). When necessary, Greenhouse–Geisser estimates of sphericity were used to correct for degrees of freedom. Bonferroni post hoc comparisons were used to evaluate group differences. For the comparisons of multiple means with genotypes as one independent variable, one-way ANOVA followed by the Tukey’s post hoc tests was used. One-sample *t* test was used to compare performance on the probe test of the Barnes maze task against chance level (25%). Data are presented as means ± SEM. All alpha levels were set at 0.05.

## Additional files

**Additional file 1: Fig. S1.** Time course of experimental procedures for assessing cognitive and emotional domains in *App*-KI mice. Based on pathological information about the brains of *App*^*NL*-*G*-*F/NL*-*G*-*F*^ mice, cognitive and emotional domains in *App*-KI mice were assessed at different ages using three behavioral assays. The same group of mice (Group 4) was assessed at 4, 6 and 8 months of age for spatial learning and memory and behavioral flexibility using the Barnes maze (BM) task, and at 15–18 months of age for contextual fear memory using the contextual fear conditioning (CFC) task. Time courses of brain pathology in *App*^*NL*-*G*-*F/NL*-*G*-*F*^ mice are shown based on previous studies.

**Additional file 2: Fig. S2.** Locomotor activity of *App*^*NL*-*G*-*F/NL*-*G*-*F*^ and *App*^*NL/NL*^ mice during the first and second trials in the elevated plus maze task. The distance travelled during the 10-min test of the first and second trials in the elevated plus maze task was compared among genotypes at both 6–9 (**a**–**d**) and 15–18 (**e**–**h**) months of age. Representative images of movement tracks during the first and second trials for each genotype at 6–9 (**a** and **c**) and 15–18 (**e** and **g**) months of age were shown (closed arms are indicated by shaded areas). At 6–9 months of age, *App*^*NL*-*G*-*F/NL*-*G*-*F*^ mice exhibited slight increases in distance travelled during the first (**b**) and second (**d**) trials in comparison with WT mice. By contrast, locomotor activity in *App*^*NL/NL*^ mice was comparable with WT mice in the two trials. At 15–18 months of age, *App*^*NL*-*G*-*F/NL*-*G*-*F*^ mice exhibited a slight increase in movement compared to WT mice during the first (**f**) and second (**g**) trials. *App*^*NL/NL*^ mice moved at similar levels compared with WT mice in the two trials. 6–9 month-old; n = 8 WT (B6J), n = 8 *App*^*NL/NL*^, n = 8 *App*^*NL*-*G*-*F/NL*-*G*-*F*^. 15–18 month-old; n = 12 WT (B6J), n = 10 *App*^*NL/NL*^, n = 11 *App*^*NL*-*G*-*F/NL*-*G*-*F*^. †*p* < 0.05 versus *App*^*NL/N*L^.

**Additional file 3: Fig. S3.** Locomotor activity in *App*^*NL*-*G*-*F/NL*-*G*-*F*^ and *App*^*NL/NL*^ mice during the pre-shock period in the contextual fear conditioning task. The distance travelled during the pre-shock period (3-min period just prior to the first footshock) in conditioning was compared among genotypes at both 6–9 (**a** and **b**) and 15–18 (**c** and **d**) months of age. Representative images of movement tracks during the pre-shock period in each genotype at 6–9 (**a**) and 15–18 (**c**) months of age were shown. At 6–9 months of age, *App*^*NL*-*G*-*F/NL*-*G*-*F*^ mice exhibited a slight decrease in distance travelled during the pre-shock period in comparison with WT mice (**b**). At 15–18 months of age, *App*^*NL/NL*^ mice exhibited a significant decrease in distance travelled during the pre-shock period in comparison with WT mice (**d**). Locomotor activity in *App*^*NL*-*G*-*F/NL*-*G*-*F*^ mice was also slightly decreased in comparison with WT mice. 6–9 month-old; n = 6 WT (B6 J), n = 6 *App*^*NL/NL*^, n = 9 *App*^*NL*-*G*-*F/NL*-*G*-*F*^. 15–18 month-old; n = 8 WT (B6 J), n = 7 *App*^*NL/NL*^, n = 7 *App*^*NL*-*G*-*F/NL*-*G*-*F*^. *p < 0.05 versus WT (B6J).

## Abbreviations

AD: Alzheimer’s disease
APP: amyloid precursor protein
Aβ: amyloid-β
NFT: neurofibrillary tangle
KI: knock-in
WT: wild-type
EPM: elevated plus maze
CFC: contextual fear conditioning
BM: Barnes maze
ANOVA: analysis of variance
CTF-β: C-terminal fragment-β
NTF-β: N-terminal fragment-β
GABA: γ-aminobutyric acid
