# Supplementary figures and images for: Cognitive and emotional alterations in App knock-in mouse models of Aβ amyloidosis
Source: BMC Neurosci. 2018 Jul 28;19:46. doi: 10.1186/s12868-018-0446-8 (PMC6064053; doi:10.1186/s12868-018-0446-8)

Brain pathology in *App<sup>NL-G-F/NL-G-F</sup>* mice

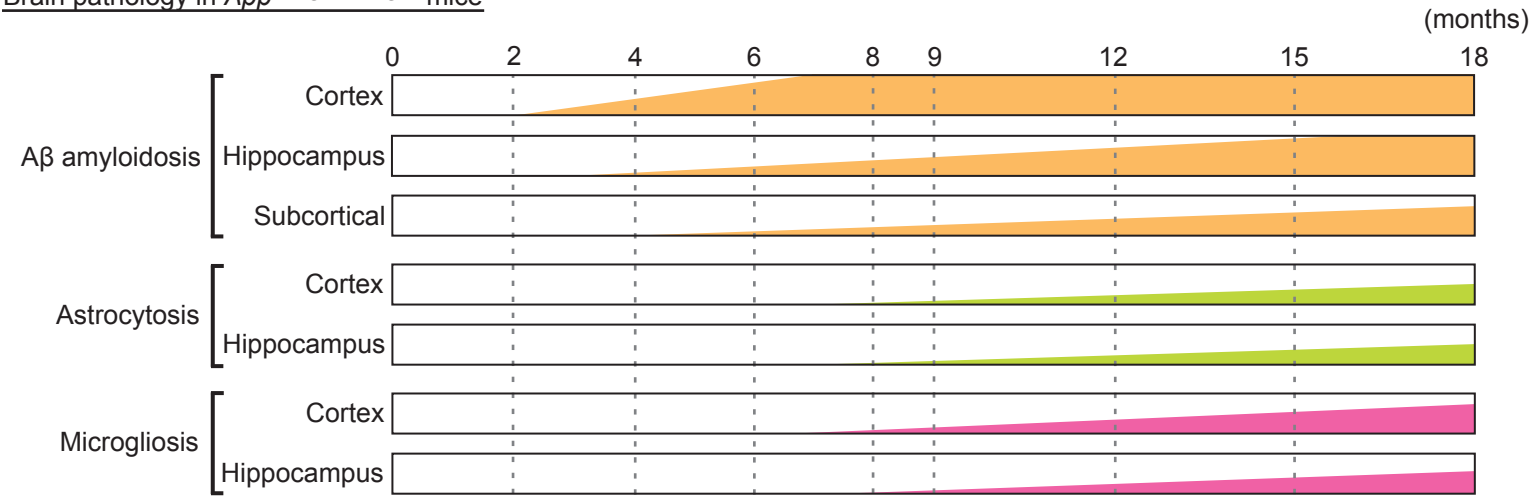

Behavioral paradigm in this study

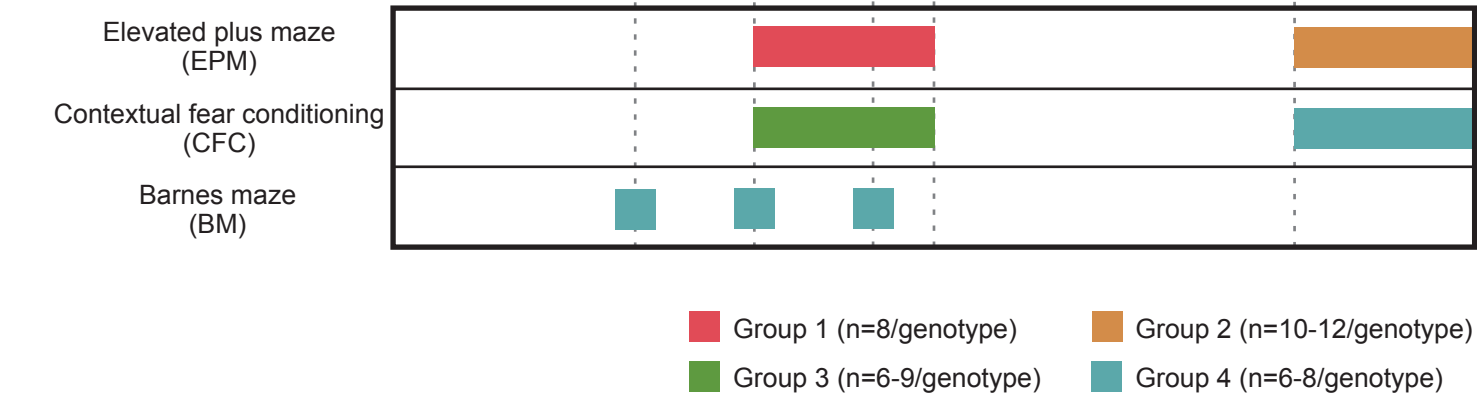

Fig. S1

Supplement: Supplementary file 1 — Additional file 1: Fig. S1. Time course of experimental procedures for assessing cognitive and emotional domains in App-KI mice. Based on pathological information about the brains of AppNL-G-F/NL-G-F mice, cognitive and emotional domains in App-KI mice were assessed at different ages using three behavioral assays. The same group of mice (Group 4) was assessed at 4, 6 and 8 months of age for spatial learning and memory and behavioral flexibility using the Barnes maze (BM) task, and at 15–18 months of age for contextual fear memory using the contextual fear conditioning (CFC) task. Time courses of brain pathology in AppNL-G-F/NL-G-F mice are shown based on previous studies. [file 12868_2018_446_MOESM1_ESM.pdf]

## 6–9 month-old

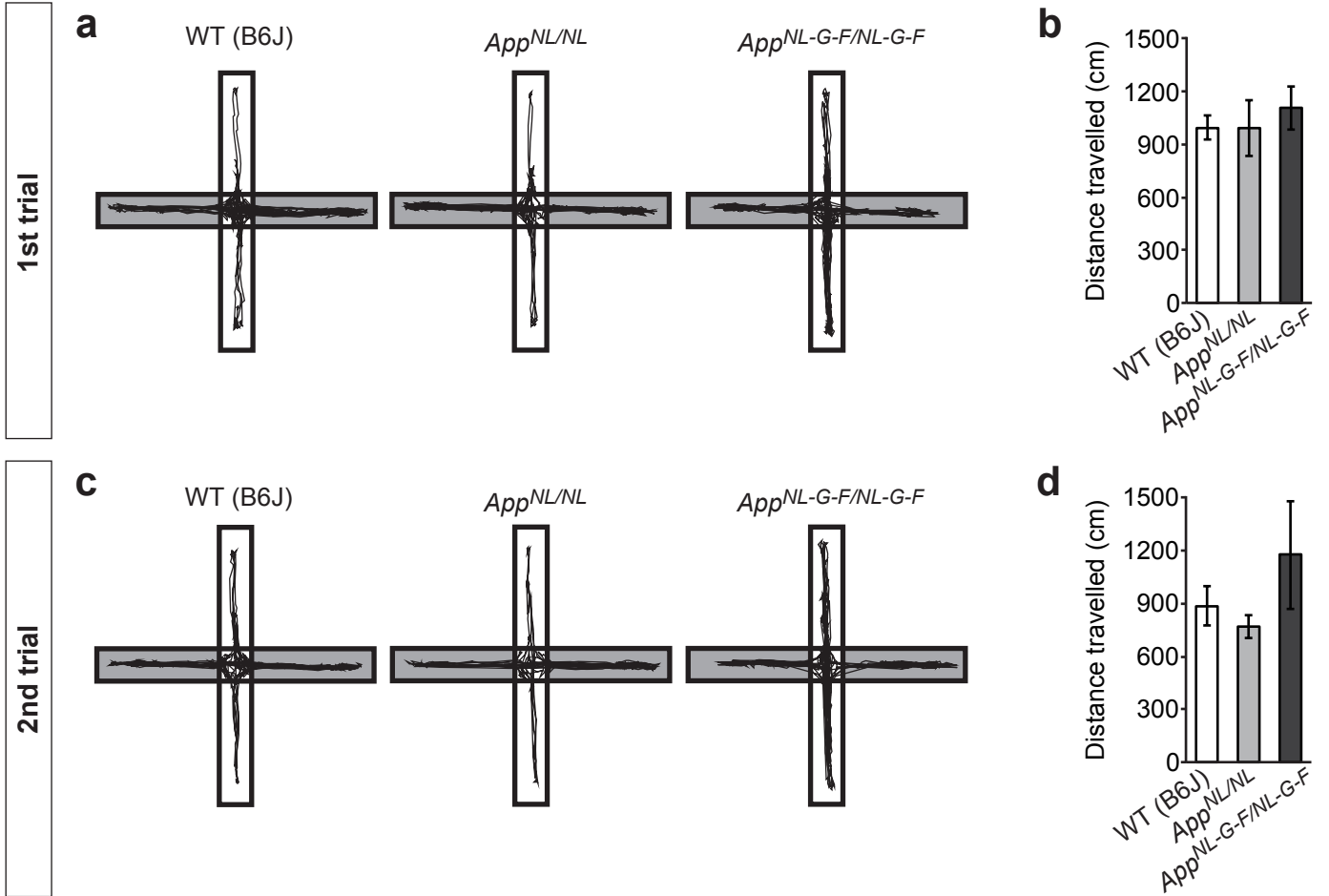

## 15–18 month-old

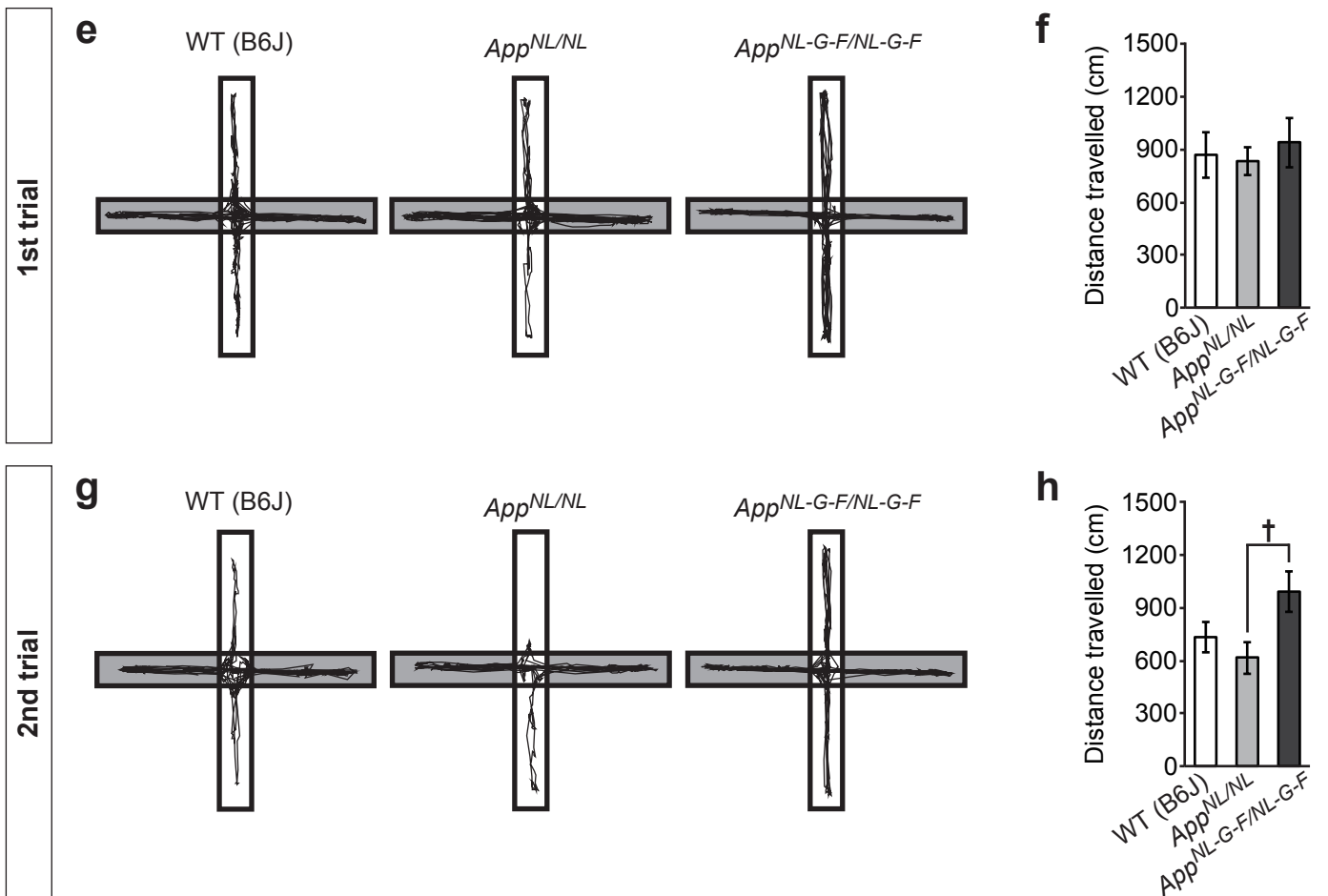

Fig. S2

Supplement: Supplementary file 2 — Additional file 2: Fig. S2. Locomotor activity of AppNL-G-F/NL-G-F and AppNL/NL mice during the first and second trials in the elevated plus maze task. The distance travelled during the 10-min test of the first and second trials in the elevated plus maze task was compared among genotypes at both 6–9 (a–d) and 15–18 (e–h) months of age. Representative images of movement tracks during the first and second trials for each genotype at 6–9 (a and c) and 15–18 (e and g) months of age were shown (closed arms are indicated by shaded areas). At 6–9 months of age, AppNL-G-F/NL-G-F mice exhibited slight increases in distance travelled during the first (b) and second (d) trials in comparison with WT mice. By contrast, locomotor activity in AppNL/NL mice was comparable with WT mice in the two trials. At 15–18 months of age, AppNL-G-F/NL-G-F mice exhibited a slight increase in movement compared to WT mice during the first (f) and second (g) trials. AppNL/NL mice moved at similar levels compared with WT mice in the two trials. 6–9 month-old; n = 8 WT (B6J), n = 8 AppNL/NL, n = 8 AppNL-G-F/NL-G-F. 15–18 month-old; n = 12 WT (B6J), n = 10 AppNL/NL, n = 11 AppNL-G-F/NL-G-F. †p < 0.05 versus AppNL/NL. [file 12868_2018_446_MOESM2_ESM.pdf]

## 6–9 month-old

**a**

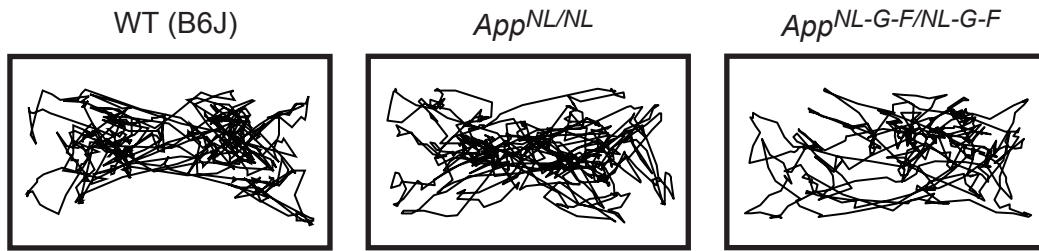

**b**

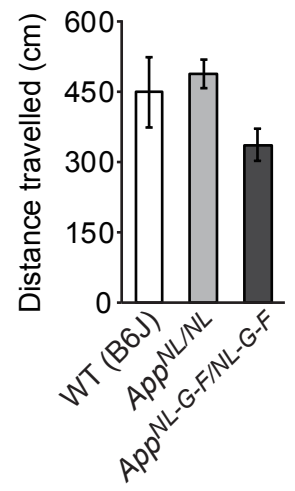

## 15–18 month-old

**c**

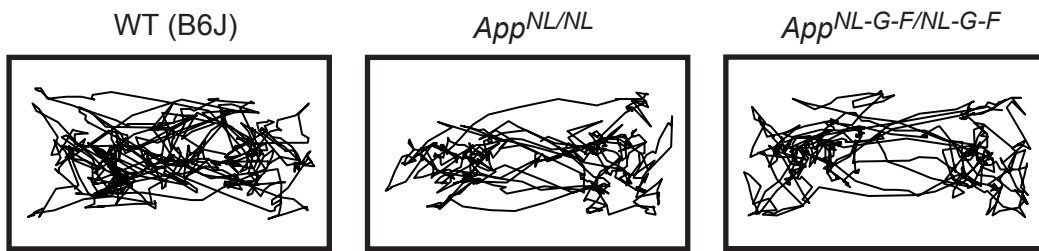

**d**

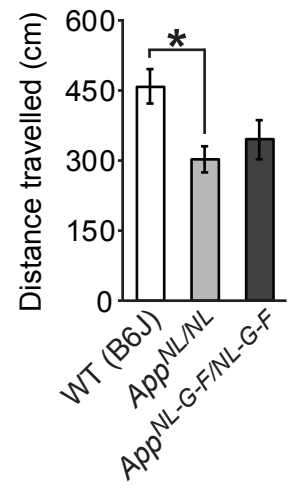

Supplement: Supplementary file 3 — Additional file 3: Fig. S3. Locomotor activity in AppNL-G-F/NL-G-F and AppNL/NL mice during the pre-shock period in the contextual fear conditioning task. The distance travelled during the pre-shock period (3-min period just prior to the first footshock) in conditioning was compared among genotypes at both 6–9 (a and b) and 15–18 (c and d) months of age. Representative images of movement tracks during the pre-shock period in each genotype at 6–9 (a) and 15–18 (c) months of age were shown. At 6–9 months of age, AppNL-G-F/NL-G-F mice exhibited a slight decrease in distance travelled during the pre-shock period in comparison with WT mice (b). At 15–18 months of age, AppNL/NL mice exhibited a significant decrease in distance travelled during the pre-shock period in comparison with WT mice (d). Locomotor activity in AppNL-G-F/NL-G-F mice was also slightly decreased in comparison with WT mice. 6–9 month-old; n = 6 WT (B6 J), n = 6 AppNL/NL, n = 9 AppNL-G-F/NL-G-F. 15–18 month-old; n = 8 WT (B6 J), n = 7 AppNL/NL, n = 7 AppNL-G-F/NL-G-F. *p < 0.05 versus WT (B6J). [file 12868_2018_446_MOESM3_ESM.pdf]
